# Theoretical and experimental analysis of the modulated phase grating X-ray interferometer

**DOI:** 10.1038/s41598-024-78133-8

**Published:** 2024-11-05

**Authors:** Hunter Meyer, Joyoni Dey, Sydney Carr, Kyungmin Ham, Leslie G. Butler, Kerry M. Dooley, Ivan Hidrovo, Markus Bleuel, Tamas Varga, Joachim Schulz, Thomas Beckenbach, Konradin Kaiser

**Affiliations:** 1 Department of Physics and Astronomy, Louisiana State University, Baton Rouge, 70803 LA USA; 2 Naval Dosimetry Center, Navy Medicine, MD 20889-5629 Bethesda, USA; 3 Center for Advanced Microstructures and Devices, Louisiana State University, Baton Rouge, 70806 LA USA; 4 Department of Chemistry, Louisiana State University, Baton Rouge, 70803 LA USA; 5 Cain Department of Chemical Engineering, Louisiana State University, Baton Rouge, 70803 LA USA; 6 Department of Radiation Therapy, Solón Espinosa Ayala Oncological Hospital, Quito, Ecuador; 7 Adelphi Technology, Inc., Redwood City, 94063 CA USA; 8 The Environmental Molecular Sciences Laboratory, Pacific Northwest National Laboratory, Richland, WA USA; 9 Microworks GmbH, Schnetzlerstr. 9, Karlsruhe, 76137 Germany; 10 Institute of Microstructure Technology, Karlsruhe Institute of Technology, Hermann-von-Helmholtz-Platz 1, Eggenstein-Leopoldshafen, D-76344 Germany

**Keywords:** X-ray interferometry, Modulated phase grating, Diffraction grating, Dark-field, Porosity, X-ray tomography, X-rays, X-rays

## Abstract

X-ray grating interferometry allows for the simultaneous acquisition of attenuation, differential-phase contrast, and dark-field images, resulting from X-ray attenuation, refraction, and small-angle scattering, respectively. The modulated phase grating (MPG) interferometer is a recently developed grating interferometry system capable of generating a directly resolvable interference pattern using a relatively large period grating envelope function that is sampled at a pitch that is small enough that X-ray spatial coherence can be achieved by using a microfocus X-ray source or G0 grating. We present the theory of the MPG interferometry system for a 2-dimensional staggered grating, derived using Fourier optics, and we compare the theoretical predictions with experiments we have performed with a microfocus X-ray system at Pennington Biomedical Research Center, LSU. The theoretical and experimental fringe visibility is evaluated as a function of grating-to-detector distance. Additionally, quantitative experiments are performed with porous carbon and alumina compounds, and the mean normalized dark-field signal is compared with independent porosimetry measurements. Qualitative analysis of attenuation and dark-field images of a dried anchovy are shown.

## Introduction

X-ray grating interferometry allows for the simultaneous acquisition of attenuation, differential-phase contrast (DPC), and dark-field images. In contrast to traditional X-ray radiography systems, one or more diffraction gratings are placed in the path of the X-ray beam so that a periodic interference pattern is produced, commonly referred to as interference fringes, which is approximately sinusoidal. With no object in the path of the X-ray beam, the interference pattern is typically referred to as the reference or *blank*image. When an object is placed in the path of the X-ray beam, it’s physical properties are imaged by measuring the perturbation to the reference fringe pattern. The pattern is perturbed in three ways, resulting in images with three distinct contrast mechanisms^1^. Attenuation causes a reduction in the average value of the fringe pattern, producing the attenuation image. Refraction results in a phase shift of the pattern, producing the DPC image. Small angle scattering reduces the fringe visibility, which is the peak-to-peak height of the fringes relative to the average value, producing the dark-field image. Interferometry has potential for a variety of applications in science and medicine, including lung imaging^2–5^, breast imaging^6–9^, arthritis imaging^10,11^, osteoporosis imaging^12^, pore size analysis^13^, additive manufacturing quality assurance^14,15^, etc.

There are presently several grating interferometers in the literature, including the Talbot-Lau Interferometer (TLI)^16–18^, Dual Phase Grating Interferometer (DPGI)^19,20^, and Modulated Phase Grating Interferometer (MPGI)^21–25^. The TLI has a phase grating, G1, that produces interference fringes that are not directly resolvable by typical detectors, meaning an analyzer grating, G2, is required to create visible Moiré patterns, resolvable at the detector. The analyzer grating’s pitch is determined by the geometric magnification of the G1 grating, meaning the geometry of the system is fixed. While this interferometer is highly sensitive due to the low-period fringes produced, the requirement of an analyzer grating, which is an absorption grating, doubles the dose per image for similar fluence at the detector. The DPGI achieves directly resolvable patterns using two phase gratings separated by a few millimeters, without the need for an analyzer grating. The Moiré pattern produced has a beat pattern directly resolvable by the detector, with the high-frequency components being washed out by blur from the detector and X-ray source. The DPGI configurations are typically far-field geometries and are often called a Far-Field interferometry systems^19^. The DPGI’s two phase gratings must be co-aligned for proper fringe formation, which often requires time-consuming alignment procedures. This problem exists for the TLI as well.

**Fig. 1:**
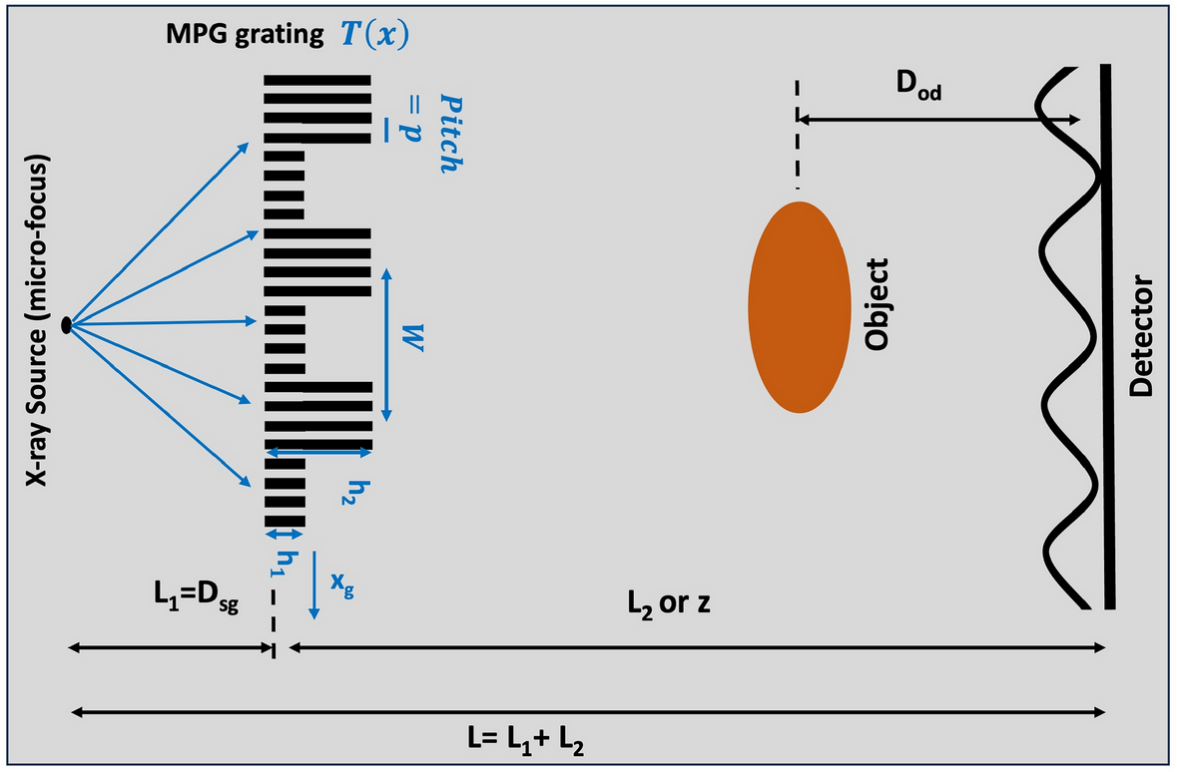
Schematic of the modulated phase grating interferometer with a micro-focus X-ray source. Here the 1-dimensional RectMPG parameters are shown, where *W* is the period of the grating’s envelope function, *p* is the grating pitch, $h_1$ and $h_2$ are the phase heights, and the geometry is defined by the source-to-grating distance, $L_1$, the grating-to-detector distance, *z*, and the object-to-detector distance, $D_{od}$. $x_g$ denotes the phase stepping direction.

The modulated phase grating interferometer (MPGI), originated by our group^21–24^, consists of a single grating where the heights of the grating bars follows an envelope function, with a relatively large period, *W*. A system diagram is shown in Figure 1. The envelope function is sampled at a high-frequency pitch, *p*, to meet the coherence requirements similar to that of the TLI and DPGI systems. In this context, the envelope period, *W*, and the sampling pitch, *p*, should not be confused, since this terminology is used interchangeably for a binary diffraction grating such as those in the TLI or DPGI systems. The fringes produced have many high-frequency harmonics that are washed out by the detector and low-frequency harmonics that result from the envelope function, analogous to the beat pattern produced by the DPGI. Images can be calculated using either a single-shot^26,27^or a phase stepping procedure^28^. While the single-shot methodology is simpler, the fringe resolution is not as high, so for this study, phase stepping was used.

A key benefit of the MPGI is the simplicity and dose efficiency of using a single grating for fringe formation. Since no analyzer grating is present, the placement of the MPG is not limited by Talbot distance constraints as in the TLI, and the dose is not doubled to a patient/object for the same attenuation image quality as a traditional X-ray. Like the DPGI, the system’s autocorrelation length can be changed by changing *either*the grating placement or patient/object placement, but since only one grating is used, grating alignment is a much simpler procedure. Another more subtle advantage is that the MPGI system is always first harmonic dominant. In contrast, the TLI and DPGI systems are often second harmonic dominant; the first harmonic disappears if they are illuminated by a monochromatic source. The first harmonic reappears when illuminated by a polychromatic source^20^, causing the interference fringes to not be as sinusoidal, leading to ambiguities in the systems’ phase sensitivity, autocorrelation length, and visibility.

In this study, we present the theory of fringe formation for the MPGI for a polychromatic X-ray source. In a previous study, we presented the theory of the 1-dimensional MPG with comparisons to simulations performed using the Sommerfeld Rayleigh Diffraction Integral (SRDI) simulator, with several orders of magnitude of decreased simulation time and comparable results^24^. In this paper, we extend the 1-dimensional MPG theory to account for the staggering of the grating bars that is implemented during fabrication for grating stability, and we compare visibility predictions with experimental measurements performed at Pennington Biomedical Research Center (PBRC) using preliminary MPGs. The purpose of these theoretical developments is to allow for the rapid simulation of modulated phase grating interferometers to aid in the design of these systems. This allows for the calculation of the fringe visibility for a wide range of system geometries, energies, and grating designs, and an analytical model of the MPG greatly reduces the amount of time for simulation, permitting rapid development of these systems.

Additionally, we present experimental imaging and analysis of a variety of samples. We have imaged multiple carbon and alumina compounds with varying porosity, and we compare the mean normalized dark-field signal with independent porosimetry measurements, showing correlations with the samples’ partial pore volume. We have also imaged a dried anchovy, and show multiple regions of interest where unique structures are seen in the dark-field images, compared with the attenuation images. The imaging of these samples show the potential for the MPGI for medical and industrial imaging applications.

## Methods

### Theory

The theory of the 1-dimensional MPG was originally derived in^24^, which will only hold for a 2-dimensional MPG if the grating is constant in one dimension. However, for this study, the MPGs used were produced by Microworks GmbH, Germany, where the grating bars were staggered for improved structural stability, following a Bridge design^29^. Here we present a revised version of the MPG theory that accounts for the staggered grating bars. We will then compare the visibility calculated using the presented theory with experimental measurements of fringe visibility for a range of system geometries.

#### A modified transmission function in 1 dimension

First, we will briefly review the theory of the 1-dimensional MPG^24^, then detail the modifications necessary for 2 dimensions. For the 1-dimensional MPG, the grating transmission function is given as,

1 $$\begin{aligned} T(x)&= \left[ g(x)\sum _{n = -\infty }^{\infty } \delta (x - np) + \sum _{n = -\infty }^{\infty } \delta (x - np - p/2) \right] \star {{\,\textrm{rect}\,}}\left( \frac{x}{\alpha p}\right) \end{aligned}$$

2 $$\begin{aligned} g(x)&= \sum _{m = \infty }^{\infty } g_m(\lambda ) \exp \left( \frac{2\pi j mx}{W}\right) \end{aligned}$$

The envelope function is represented by *g*(*x*) and is periodic. It can be represented as a Fourier Series of period *W*, with Fourier coefficients, $g_m$. In Equation 1, the first series of $\delta$ functions samples the envelope function at a sampling period of *p*, and the convolution with the ${{\,\textrm{rect}\,}}$ function places a grating bar at each sample, since $f(x) \star \delta (x - x_0) = f(x - x_0)$, where $\star$ represents the convolution. $\alpha$ represents the duty cycle of the grating and *j* is the imaginary number. The second series of $\delta$ functions is responsible for the gaps between the grating bars, which is necessary for the grating transmission to equal 1, not 0.

The envelope function’s Fourier coefficients, $g_m$, are energy-dependent and account for the transmission function’s amplitude reduction and phase change. The physical heights of the grating bars are designed to follow the desired phase envelope at the design energy, $E_D$. The grating attenuation is determined by the physical heights of the grating bars resulting from that design. The attenuation and phase profile of the grating are energy-dependent, therefore we represent the grating coefficients as $g_m(\lambda )$. The polychromatic intensity is simply the incoherent superposition of each monochromatic intensity weighted by the energy spectrum. This means the energy spectrum affects the detector intensity in two ways: by diffraction and by the grating coefficients. The field amplitude can be derived using the angular spectrum method under the Fresnel approximation^30^, and the detector intensity can be calculated as simply the square of the field amplitude, $I(x,z) = |U(x,z)|^2$.

It is difficult to generalize Equation 1 to 2 dimensions, since defining the regions between the grating bars becomes cumbersome. Instead, an equivalent transmission function, written in another form, is introduced. Here, the grating bars result from sampling, and a constant transmission of 1 is included to account for the regions between the grating bars. This constant transmission is then subtracted from *g*(*x*) so that the transmission of the grating bars follows the envelope function. The equivalent transmission function in 1 dimension is shown in Equation 3. It is straightforward to show that this function is exactly the same as Equation 1.

The new transmission function is used to derive the field amplitude shown in Equation 4using the angular spectrum method^30^. Though there are notational differences, the calculated field amplitude is exactly the same as what is found in our previous work^24^ (using the first transmission function, Equation 1).

3 $$\begin{aligned} T(x)&= 1 + \left[ (g(x) - 1) \sum _{n = -\infty }^{\infty } \delta (x - np) \right] \star rect\left( \frac{x}{\alpha p}\right) \end{aligned}$$

4 $$\begin{aligned} U(x,z)&= 1 + \alpha \sum _m \sum _n b_1(m, n, z) \exp \left( j 2 \pi x \left( \frac{m}{W} + \frac{n}{p} \right) \right) - \alpha \sum _n b_2(n, z) \exp \left( j 2 \pi x \left( \frac{n}{p}\right) \right) \end{aligned}$$

5 $$\begin{aligned} b_1(m,n, z)&= g_m {{\,\textrm{sinc}\,}}\left( \alpha p \left( \frac{m}{W} + \frac{n}{p} \right) \right) \exp \left( -j \pi \lambda z \left( \frac{m}{W} + \frac{n}{p} \right) ^2 \right) \end{aligned}$$

6 $$\begin{aligned} b_2(n, z)&= {{\,\textrm{sinc}\,}}\left( \alpha n\right) \exp \left( -j \pi \lambda z \left( \frac{n}{p}\right) ^2\right) \end{aligned}$$

**Fig. 2:**
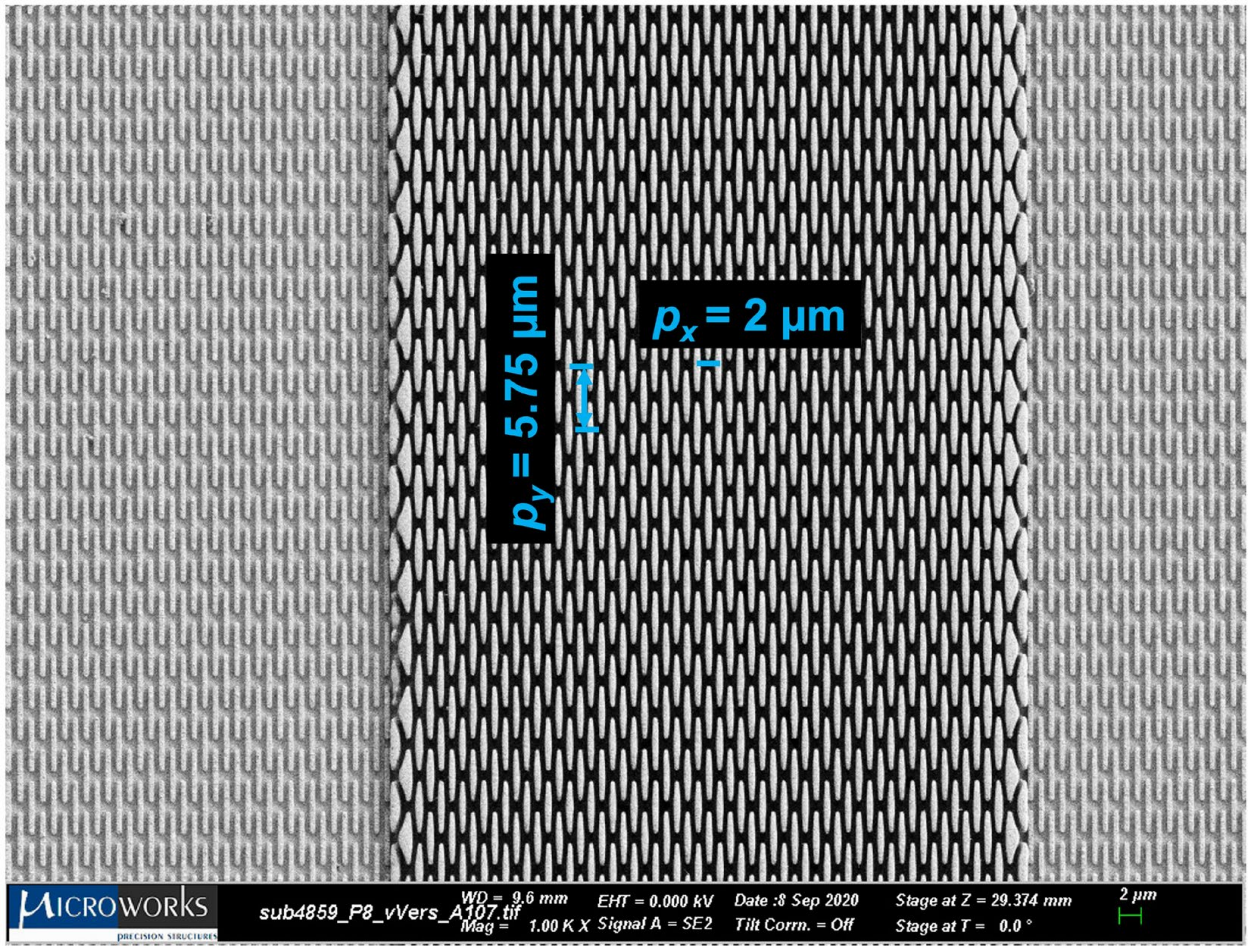
An SEM image of the staggered 2D modulated phase grating, with $p_x$ and $p_y$ labeled. $\alpha _x$ and $\alpha _y$ are the duty cycles of the grating in the x- and y-dimension, respectively.

#### The 2-dimensional intensity calculation

The fringe pattern created by a 2-dimensional modulated phase grating must consider the effect of the Bridge design^29^, where the grating bars are staggering for improved structural stability. A microscopic image of one of the gratings used in this study is shown in Figure 2. To incorporate the staggered grating bars into the MPG theory, the X-ray transmission function must be adapted to two-dimensions, shown in Equation 7. A noticeable change from Equation 3 is the reintroduction of a second sampling function (not to be confused with the double sampling in Equation 1, since both sampling functions follow *g*(*x*)), which is responsible for the vertical staggering of the grating bars. Additionally, there is now a pitch in each direction, $p_x$ and $p_y$, as well a duty cycle in each direction, $\alpha _x$ and $\alpha _y$. Notice that in the context of the 2D grating, the pitch is the distance to *every other* grating bar, and the duty cycle is the width (in x or y) of the bar relative to the pitch. Here, the two sampling terms both follow *g*(*x*) but are offset by $p_x/2$ and $p_y/2$, to account for the staggering of the grating bars. Note that if $\alpha _y = 1$, $\alpha _x = \alpha /2$, and $p_x = 2 p$, the staggering will be completely removed, and the 2D transmission function will be equivalent to the 1D transmission function.

7 $$\begin{aligned} T(x,y)= & 1 + \Bigg \{ (g(x) - 1) \biggl [\sum _n \sum _l \delta (x - n p_x)\delta (y - l p_y) + \sum _n\sum _l \delta \left( x - n p_x - \frac{p_x}{2} \right) \delta \left( y - l p_y - \frac{p_y}{2} \right) \biggr ] \Bigg \} \nonumber \\ & \star rect \left( \frac{x}{\alpha _x p_x}\right) rect \left( \frac{y}{\alpha _y p_y}\right) \end{aligned}$$

The derivation of the diffracted intensity is quite lengthy, so it is placed in the Supplementary Material. The field amplitude is derived using the angular spectrum method^30^, followed by the Fresnel scaling theorem^31^ to scale the intensity from that of a plane-wave to that of a point-source. The intensity at the detector is calculated as

8 $$\begin{aligned} I(x,y,z) = 1 + \sum _l \biggl [ c(l,x,z) + c^*(-l,x,z) + d(l,x,z) \biggr ] \exp \left( j 2 \pi y \frac{l}{M p_y} \right) \end{aligned}$$

where *c*(*l*, *x*, *z*) is defined in Supplementary Equation S22 and $d(l,x,z) = c(l,x,z) \star c^*(-l,x,z)$. Here only the y-harmonics are shown, but the x-harmonics are contained within *c*(*l*, *x*, *z*) and *d*(*l*, *x*, *z*).

Next, as detailed in the Supplementary Material (Equations S27-S28), to simplify calculations, the 2D intensity is approximated by the $l = 0$ harmonic, where *l* represents the harmonic number in the y-dimension. *This means that we keep only the zero-th harmonic (mean) in the y-direction*. This approximation is valid when the detector and source blur are sufficiently high enough to blur the $l \ne 0$ y-harmonics that result from the staggering of the grating bars necessary for fabrication stability. Notably, while this approximation removes the dependence of $p_y$, *c*(*l*, *x*, *z*) and *d*(*l*, *x*, *z*) still depend on the duty cycle in the y-direction, $\alpha _y$. The staggering of the grating bars still affects the fringe visibility, even when there is sufficient blur to approximate the 2-dimensional intensity profile into a single dimension.

9 $$\begin{aligned} I(x,y,z) \approx 1 + c(0, x, z) + c^*(0, x, z) + d(0, x, z) \end{aligned}$$

Supplementary Equation S27 is the intensity calculated under the $l = 0$ Approximation and is reproduced in Equation 9. *The* $l = 0$ *approximation provides a powerful tool to make 2D intensity calculations fast under realistic conditions when the detector point spread function (PSF) and source blur combination is larger than the (magnified) grating pitch in the y-dimension*, $p_y$. This approximation greatly reduces the computation required, since only one dimension needs to be calculated, instead of two.

#### Post-processing and visibility calculations

The intensity shown in Equation 9 is the intensity profile when the source is a monochromatic point source. To properly model the fringe profile and visibility measured in an experiment, we must include a polychromatic source, finite focal spot size, and detector point spread function. Additional post-processing includes modeling the phase stepping procedure and downsampling to the detector sample rate.

The intensity of a polychromatic point source can be found by integrating over the energy spectrum, *S*(*E*).

10 $$\begin{aligned} I_{poly}(x,y,z) = \int _E I(x,y,z; E) S(E) dE \end{aligned}$$

The effect of finite focal spot size can be found by convolving the point-source intensity profile with the magnified source profile, *s*(*x*). It’s important to note that the source is magnified by a factor of $M - 1 = \frac{z}{L1}$, as shown in Appendix B of^24^,

11 $$\begin{aligned} I_{poly, spot}(x,y,z) = I_{poly}(x,y,z) \star s\left( \frac{x}{M-1}\right) \end{aligned}$$

Finally, the detector point spread function (PSF) is convolved to get the final intensity profile,

12 $$\begin{aligned} I_{final}(x,y,z) = I_{poly, spot}(x,y,z) \star PSF(x,y) \end{aligned}$$

The phase stepping procedure is modeled by shifting the intensity profile by the phase step size, $x_g$, multiplied by the magnification factor, *M*. Following this, downsampling is performed to match the detector pixel size. The intensity is initially calculated at a sample rate much higher than the pixel size. For the purposes of this study, the intensity profile is initially calculated at a rate of $0.1 \, \mu m$, and the Dexela 1512 detector used in this study has a pixel size of $75 \, \mu m$. Additionally, in the theoretical simulations, downsampling should occur *after*phase stepping. The visibility is measured following the methods of Marathe et al^28^..

#### Theory validation

The presented theory is evaluated in several ways in the Results (Theory). The intensity profile calculated for the 2D MPG under the $l = 0$ approximation is shown to be equivalent to a full 2D MPG simulation with sufficient blur in the y-direction and a sufficiently small $p_y$. It is also shown that the $l = 0$ approximation is further reduced to the true 1D MPG—where the staggering is removed and it is constant in one dimension and the field amplitude is shown in Equation 4—under the conditions of $\alpha _x = \alpha /2$, $p_x = 2p$, $\alpha _y = 1$.

Additionally, several grating designs are simulated with our experimental conditions, where a fixed source-to-detector distance of $L = 110 \, cm$ is used with a $45 \, kVp$ microfocus source, with results shown in Theoretical Visibility. The first is the case of the $(\pi , 0)$RectMPG, which are the ideal phase heights for maximum visibility^24^. However, the MPGs used in our experiments were designed to have phase heights of $(\pi /2, \pi /8)$, as shown in Table 1. We compare the theoretical visibility produced the two designs. We also compare the theoretical visibility produced by phase heights that match the design (Table 1) to the theoretical visibility produced using the measured heights of our gratings, shown in Table 2.

### Experimental methods

Several experiments were performed in the Keck Imaging Laboratory at Pennington Biomedical Research Center (PBRC) for the purposes of this study. Two preliminary MPGs were used, both with rectangular phase modulation and the same design parameters, listed in Table 1. The gratings, referred to as MPG7 and MPG8, were manufactured by Microworks, GmbH. They provided the measured heights of the grating structures, listed in Table 2, where it is seen that MPG8 has a larger difference in the heights than the design, and MPG7 has a lower difference than the design. Due to the novelty of the grating fabrication process, the height of the grating structures ($h_1$ and $h_2$) is not highly reproducible, which can explain the observed differences. A Hamamatsu L9181-02 microfocus X-ray tube was used with a Dexela 1512 X-ray Detector. The X-ray source was consistently run at $45 \, kVp$ and $55 \, \mu A$, under the small focus spot mode ($5 - 8 \, \mu m$). The system had a fixed source-to-detector distance of $L = 110 \, cm$.

**Table 1:** Modulated phase grating parameters used in visibility measurements. $h_1$ and $h_2$ are listed as their corresponding phase-shift in radians.

MPG Parameter	MPG7/MPG8
$\varvec{W \, (\mu m)}$	120
$\varvec{p_{x} \, (\mu m)}$	2
$\varvec{p_{y} \, (\mu m)}$	5.75
$\varvec{\alpha _{x}}$	0.25
$\varvec{\alpha _{y}}$	0.86
**Design** $\varvec{h_{1}}$	$\pi / 8$
**Design** $\varvec{h_{2}}$	$\pi / 2$
**Modulation Duty Cycle**	0.5
**Material**	Gold (Au)

**Table 2:** Comparison of the average measured height of the grating structures, $h_1$ and $h_2$, with the designed heights, for MPG7 and MPG8. Uncertainties shown are the standard deviation. The heights were measured by the manufacturer, Microworks, GmbH.

	Design $\varvec{\phi }$	Design Height $\varvec{(\mu m)}$	MPG7 Height $\varvec{(\mu m)}$	MPG8 Height $\varvec{(\mu m)}$
$\varvec{h_{2}}$	$\pi / 2$	2.41	$2.17 \pm 0.57$	$2.54 \pm 0.42$
$\varvec{h_{1}}$	$\pi / 8$	0.602	$0.69 \pm 0.17$	$0.62 \pm 0.18$
$\varvec{\Delta h}$	$3\pi /8$	1.81	1.48	1.88

#### Visibility measurements

The visibility was measured as a function of grating-to-detector distance, *z*, for a fixed source-to-detector distance, $L = 110 \, cm$, for both MPG7 and MPG8. The grating-to-detector distance ranged from $56 - 90 \, cm$ in $2 \, cm$ increments. The grating was phase stepped 7 times, at $30 \, \mu m$increments and with 20 second exposures, and the visibility was measured using the methods of Marathe et al^28^. The experimentally measured visibility is compared with the theoretical visibility calculated using the measured phase heights shown in Table 2, shown in Experiment Results.

For the purposes of comparing the experimentally acquired visibility with that predicted by the presented theory, the detector PSF was measured by imaging the TO MTF tungsten edge phantom by Leeds Test Objects^32^ at a small angle close to the detector. A forward model was produced of an angled edge and a generalized Gaussian was fit to find the blur induced by the detector, yielding a PSF with generalized Gaussian parameters of $\sigma = 57.87 \, \mu m$ and $k = 0.9$. The source profile was measured following the methods of Nishiki et al^33^., again using the TO MTF phantom, yielding a Gaussian source profile with $\sigma = 3.14 \, \mu m$.

#### Carbon and alumina samples: image acquisition and analysis

Images of several porous carbon and alumina samples were taken using MPG7, over an autocorrelation length (ACL) range of approximately $20 - 80 \, nm$ for the purposes of measuring how the dark-field signal changed as a function of ACL. This was achieved by taking one set of images with a source-to-grating distance of $L_1 = 20 \, cm$ and grating-to-object distances of $D_{GO} = 10, \, 25, \, 40, \, 55 \, cm$ and another set with $L_1 = 30 \, cm$ and $D_{GO} = 10, \, 20, \, 30, \, 40 \, cm$. The ACL was calculated for each geometry using a $25 \, keV$ peak energy using Equation 13, where $p_D$ is the fringe period at the detector. For each acquisition, the MPG was phase stepped 24 times, at $12 \, \mu m$ increments and with 20 second exposures.

13 $$\begin{aligned} ACL = \lambda \frac{D_{OD}}{p_D} \end{aligned}$$

Three carbon samples and three alumina samples were used for this study. The first two carbon samples were commercially available adsorbent carbons known as Nuchar (Westvaco) and Calgon-PCB that are used for water and air purification and odorant removal. The third carbon sample is an organic mesoporous carbon (OMC) produced internally that is used as a catalyst support. We refer to this compound as OMC-6-600, because it was calcined in flowing $N_2$ at $600 ^\circ \, C$. Each carbon sample was in the form of a powder, so images were acquired with each sample in a plastic capillary tube. For the alumina samples, two commercial gamma aluminas were used, known as ASM-385 and SAS-90, also used as catalyst supports. The third alumina sample was a commercially available silica-alumina porosimetry reference material made of approximately 12-14% alumina. ASM-385 and SAS-90 were in the form of compact spheres larger than a capillary tube, so they were placed in a larger plastic tube for imaging. The silica-alumina was in the form of tiny, approximately cylindrical particles and was also placed into a larger plastic tube. Because of the relative size difference in the size of the cylinders and the tube, some overlap was inevitable over the path of the ray.

For the carbon samples, a tall rectangular ROI was chosen in the center of each tube, to approximate constant thickness through the path of X-ray. For the alumina spheres, a small square ROI was chosen at the center of a single sphere, again to approximate constant thickness. The same sphere was followed for each image, to avoid minor variations between spheres. For the silica-alumina, overlap was inevitable due to the size of the cylinders relative to their container, but approximately the same ROI was chosen for each geometry.

For each ROI, the attenuation and dark-field signals were averaged to be plotted versus ACL. Generally speaking, the attenuation signal is expected to not change due to the geometry, but the dark-field signal may change as the ACL is changed, depending on the size and shape of the scattering structures within the sample. Additionally, the mass attenuation coefficient, $\mu / \rho$, will be approximately constant within each set of carbon and alumina samples. This is clearly true for the carbon samples, but for the set of alumina samples there is more nuance. The pure alumina samples (ASM-385 and SAS-90) obviously have the same mass attenuation coefficient, but this is not obvious for the silica-alumina compound. However, according to NIST’s XCOM database^34^, silica and alumina have approximately the same $\mu / \rho$, meaning the compound also has approximately the same mass attenuation coefficient as the other alumina samples.

However, their mass thickness, $\rho t$, varies due to the differences in the packing density (for the carbon samples), the porosity differences between the samples, and the pixel-to-pixel thickness variation (however small). This can be corrected if we normalize the log-scale dark-field image by the log-scale attenuation image. Assuming the mass attenuation coefficient, $\mu / \rho$, is approximately constant over the projection of one pixel, the attenuation contrast can be represented as Equation 14. The dark-field contrast can be represented in a similar manner as shown in Equation 15, using the linear diffusion coefficient, $\epsilon / \rho$, which depends on the pore microstructures within the sample. Thus, normalizing the dark-field signal by the attenuation signal corrects for the mass thickness, $\rho t$, and all that is left is the linear diffusion coefficient scaled by the mass attenuation coefficient. We call this the normalized dark-field image.

14 $$\begin{aligned}&-\ln \left( \frac{a_{0,sample}}{a_{0,blank}} \right) = \frac{\mu }{\rho } \times \rho t \end{aligned}$$

15 $$\begin{aligned}&-\ln \left( \frac{V_{sample}}{V_{blank}} \right) = \frac{\epsilon }{\rho } \times \rho t \end{aligned}$$

#### Carbon and alumina samples: porosimetry

Independent porosimetry measurements of each sample were acquired using an ASAP 2020 Plus porosimeter. Nitrogen adsorption-desorption measurements were taken, and the differential pore volume distribution with respect to pore diameter, *dV*/*dD*, was computed by the Barrett-Joyner-Halenda (BJH) algorithm^35^. From this, the total pore volume can be calculated by integrating the differential pore volume with respect to pore diameter. Since our interferometer was only sensitive to pores around the ACL at acquisition, we limited the integration range to only pores between $10 - 120 \, nm$ and called it the partial pore volume (PPV). For the purposes of calculating the PPV, BJH adsorption measurements were used for the carbon samples while BJH desorption was used for the alumina samples, due to the differences in pore *shapes*commonly seen between these samples^36,37^.

Since the mass attenuation coefficient is expected to be the same for the three carbon samples and the same for the three alumina samples, we expect the average normalized dark-field signal to follow the trend of the partial pore volume, even when the dark-field contrast curve without normalization may not. However, the two sets of samples cannot be quantitatively cross-compared, since the mass attenuation coefficient of the carbon samples is different from the alumina samples.

#### Anchovy imaging

For the purpose of showing the MPGI’s potential for biological imaging, we have imaged a dried anchovy. An anchovy was chosen because it was small enough to image with our preliminary MPGs and because of the thin bony structures within its body. The anchovy was imaged using MPG7, with the source operated at $45 \, kVp$, and a source-to-detector distance of $L = 110 \, cm$ and a source-to-grating distance of $L_1 = 20 \, cm$ were used. The anchovy was placed $13.5 \, cm$ behind the grating, providing an autocorrelation length of $ACL = 57.5 \, nm$. This geometry was chosen to maximize the field of view over the anchovy, since MPG7 is $5 \, mm \text { by } 5 \, mm$.

## Results

### Theory results

The theory presented in Methods is evaluated in several ways. The intensity profile is calculated for the 2D MPG, and the 2D MPG under the $l = 0$ approximation is compared to the case with sufficient detector blur in the y-direction and a sufficiently small $p_y$. The $l = 0$ approximation is then compared to the 1D MPG under the conditions of $\alpha _x = \alpha /2$, $p_x = 2p$, $\alpha _y = 1$, which corresponds to the removal of the staggering of the grating bars. Lastly, the visibility as a function of grating-to-detector distance, *z*, is calculated for an *ideal* MPG with $(\pi , 0)$ phase heights, an MPG with phase heights that match the design of MPG7 and MPG8, $(\pi /2, \pi /8)$, and MPGs that match the physical heights of the grating bars, as shown in Table 2. The same setup conditions from our experiments were used in our simulations.

#### The validity of the $l = 0$ approximation

**Fig. 3:**
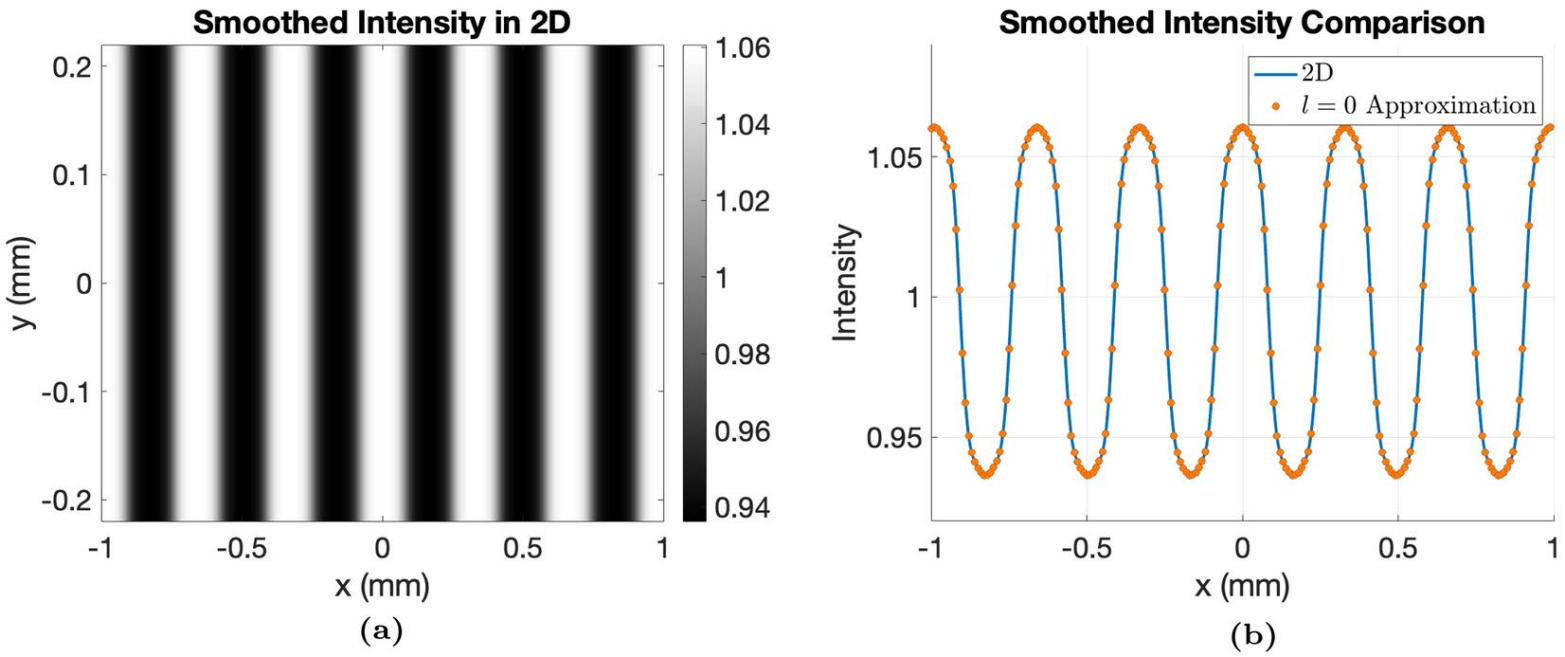
Verification of the $l = 0$ approximation using a RectMPG (Table 1) at $25 \, keV$, $L = 110 \, cm$, $z = 70 \, cm$. (**a**) Theoretical 2D intensity calculation after smoothing (**b**) Overlay of a single line through the smoothed 2D intensity profile and the $l = 0$ Approximation.

The $l = 0$ approximation is validated by showing that the analytical 2D field calculations are well approximated by the $l = 0$ approximation when the blur in the y-dimension is sufficiently high. The 2D field amplitude at the detector is found from Supplementary Equation S6. The intensity is then calculated as $I = |U(x,y,z|^2$. A monochromatic case of $25 \, keV$ is simulated for a realistic MPG with a rectangular envelope with a source-to-detector distance of $L = 110 \, cm$ and grating-to-detector distance of $z = 70 \, cm$. A realistic Gaussian source with $\sigma = 3.14 \, \mu m$ and generalized Gaussian PSF with $\sigma = 57.87 \, \mu m$ and $k = 0.9$ were used. These parameters were determined experimentally for the Keck imaging system, as explained in Experimental Methods, leading to Figure 3a. Under the same conditions, the intensity using the $l = 0$ approximation from Equation S27 is calculated and is overlaid in Figure 3b. There is excellent agreement between the blurred fully calculated 2D intensity and the intensity calculated using the $l = 0$ approximation, verifying that this approximation is appropriate for realistic MPG and setup conditions since the source and detector blur in the y-dimension is sufficiently high enough to remove the other harmonics. The $l = 0$ approximation required about 3000 times less computations for a sample rate of $0.1 \, \mu m$ and a vertical field-of-view (FOV) of $0.3 \, mm$. This FOV was necessary for the convolution of the Gaussian source (magnified) and detector PSF over 3 standard deviations.

**Fig. 4:**
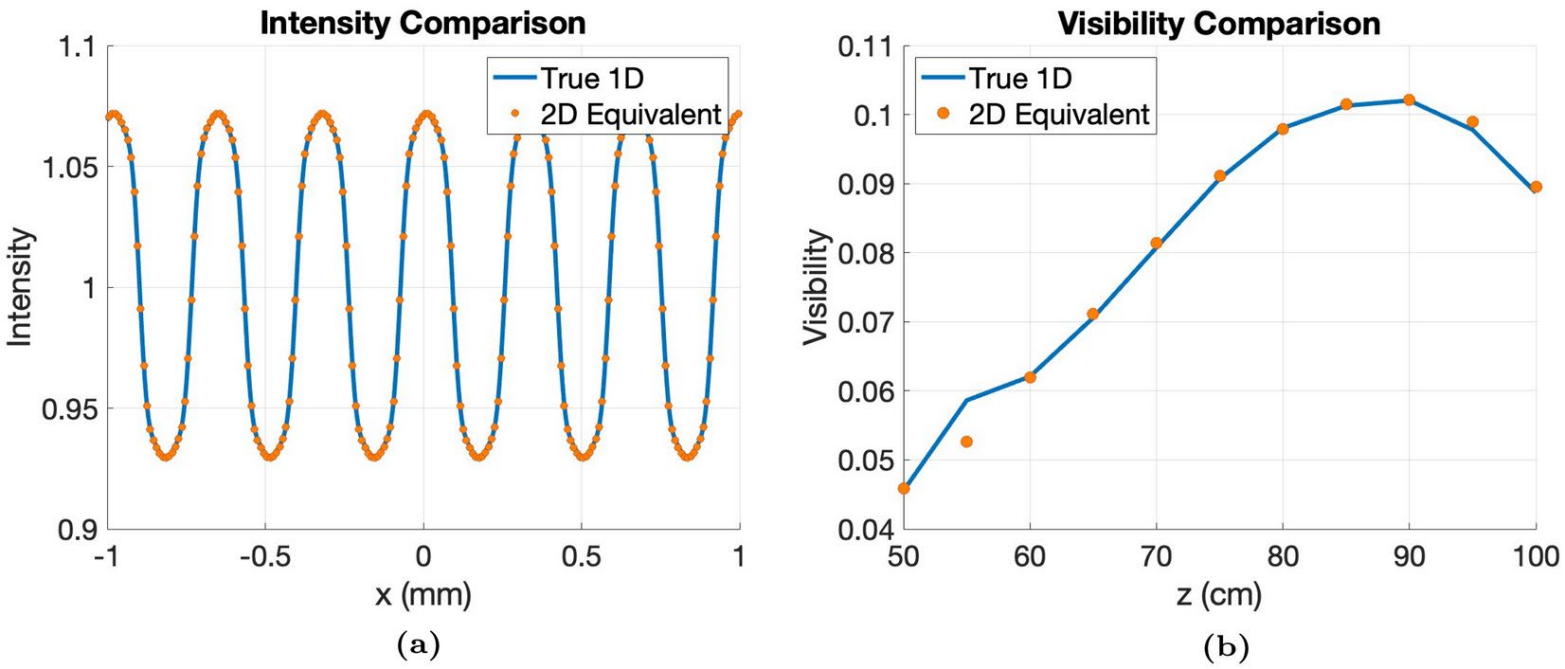
Verification of the $l = 0$ approximation with the equivalent 1D RectMPG with parameters $\alpha = 0.5$, $p = 1 \, \mu m$, $W = 120 \, \mu m$, $h_2 = \pi / 2$, $h_1 = \pi / 8$, $E_D = 25 \, keV$, made of Gold. Calculated using a monochromatic $25 \, keV$ source, a fixed source-to-detector distance of $L = 110 \, cm$, and realistic source and detector blur (**a**) Overlay of the 1D intensity with the 2D equivalent found using the $l = 0$ approximation (**b**) Visibility versus grating-to-detector distance comparison between the true 1D and 2D equivalent found using the $l = 0$ approximation.

Furthermore, as seen in Figure 4, our methodology is shown to be consistent with the 1D MPG from previous work^24^, when $\alpha _y = 1$, $p_x = 2p$ and $\alpha _x = \alpha / 2$ where the 1D pitch is *p* and $\alpha$ is the grating’s duty cycle. Over a range of geometries, the fringe profile of the true 1D MPG was calculated using Equation 4, and the fringe profile of the equivalent 2D MPG was calculated using the $l = 0$ approximation, Equation S27. The visibility was calculated as a function of grating-to-detector distance, *z*, for a fixed source-to-detector distance, *L*, and good agreement was shown between the two methods. It is evident that the 2D MPG is reduced to the 1D MPG when the staggering of the grating bars is removed when $\alpha _y = 1$ and by scaling $p_x$ and $\alpha _x$ to the equivalent 1D MPG parameters.

#### Theoretical visibility

**Fig. 5:**
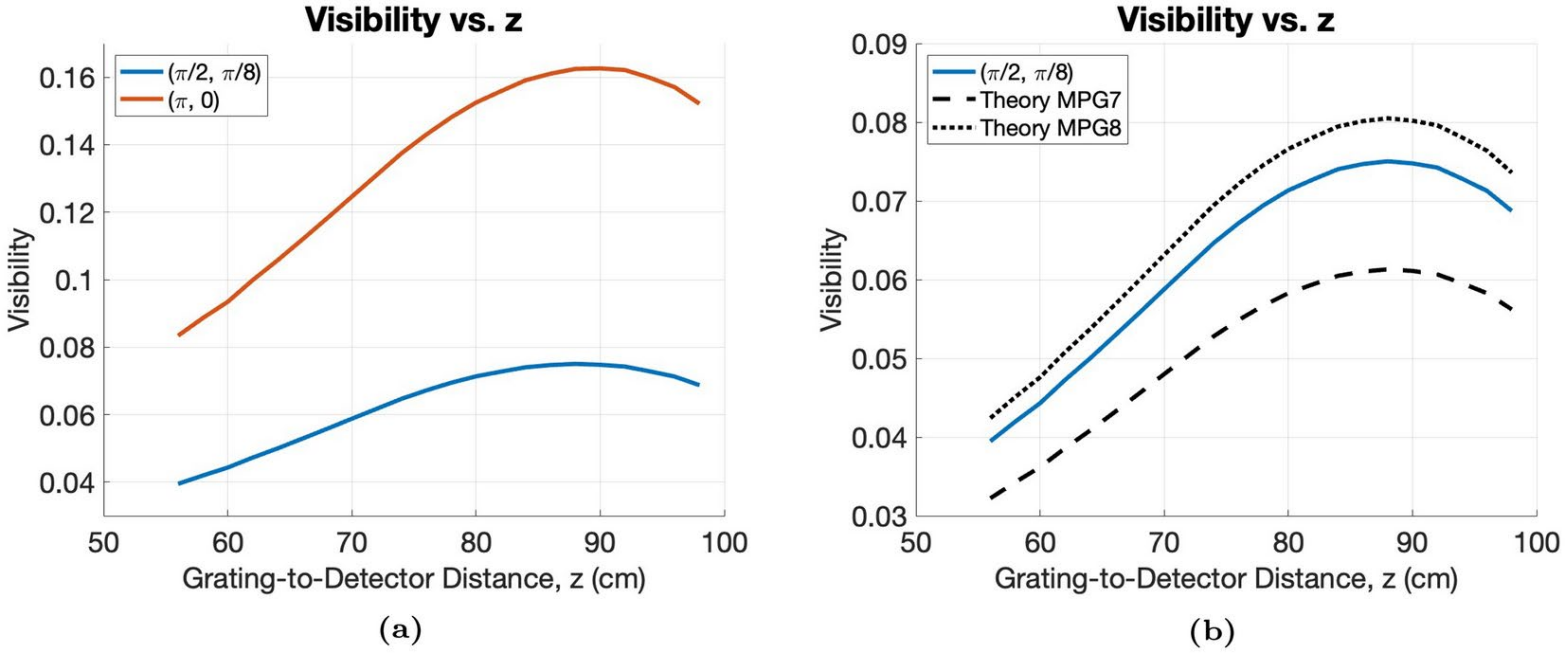
Theoretical visibility vs. grating-to-detector distance, *z*, for (**a**) an ideal $(\pi , 0)$ RectMPG and an ideal $(\pi /2, \pi /8)$ RectMPG and (**b**) an ideal $(\pi /2, \pi /8)$ RectMPG compared with theoretical simulations of the measured heights of MPG7 and MPG8, with heights shown in Table 2. All parameters are the same between the simulations except for the phase heights of the grating envelope function, $h_2$ and $h_1$.

Additional simulations were performed for multiple MPGs placed in geometries that matched our experimental visibility measurements. These include the ideal case of the $(\pi , 0)$ RectMPG, a RectMPG with heights that match the original design of MPG7 and MPG8, $(\pi /2, \pi /8$, and RectMPGs that match the measured heights of MPG7 and MPG8 shown in Table 2. The results are shown in Figure 5, where it is seen that the $(\pi , 0)$ RectMPG has higher visibility than the $(\pi /2, \pi /8)$ RectMPG at all distances, and MPG8 produces a visibility that is higher than the design and MPG7 produces a visibility that is lower than the design, due to the differences in the physical heights of the grating bars compared with the original design, as shown in Table 2.

### Experiment results

#### Experimental visibility compared with theory

**Fig. 6:**
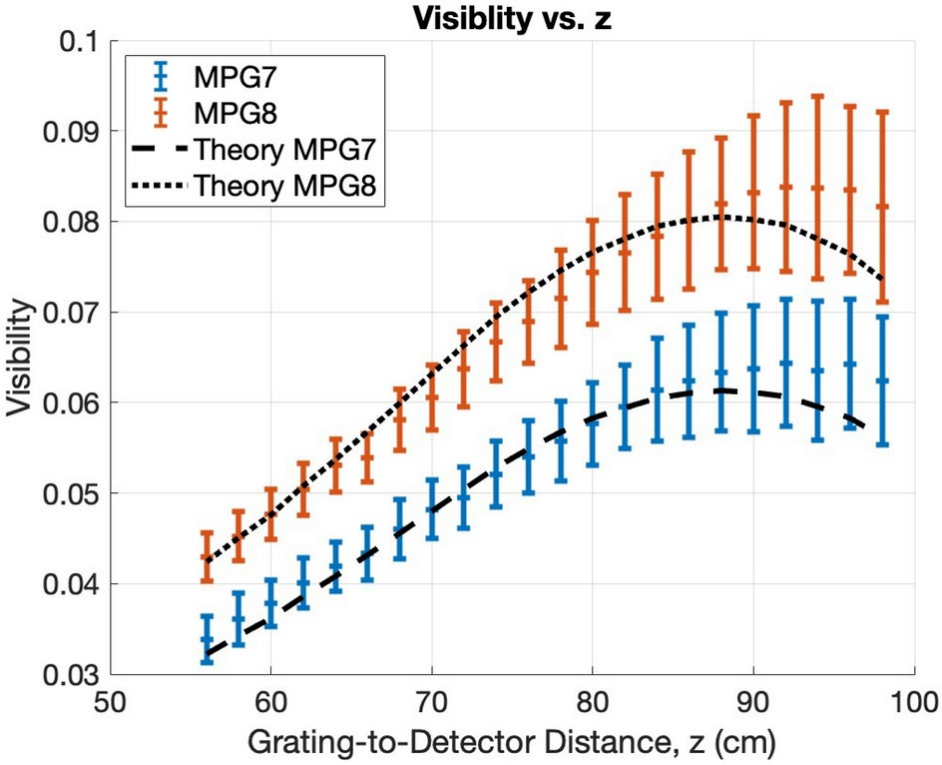
Comparison of visibility vs. grating-to-detector distance, *z*, for MPG7, MPG8, and the presented theory. The visibility was measured as as function of grating-to-detector distance, *z* for a fixed source-to-detector distance, $L = 110 \, cm$. Notice that the two theory lines are the same as in Figure 5b. The experimental visibility was calculated near the center of the grating and the mean is shown. The error bars represent the standard deviation.

The visibility produced by MPG7 and MPG8 was experimentally measured as a function of grating-to-detector distance, *z*, for a fixed source-to-detector distance, $L = 110 \, cm$, as described in Experimental Methods (Visibility Measurements). The results are shown in Figure 6, and the theoretical visibility that was simulated using the measured phase heights from Table 2 is overlaid. It is seen that MPG8 produces a higher visibility than MPG7, due to the higher phase heights, and the theoretical predictions are comparable, falling within one experimental standard deviation at all distances.

#### Carbon and alumina samples: porosimetry results

**Fig. 7:**
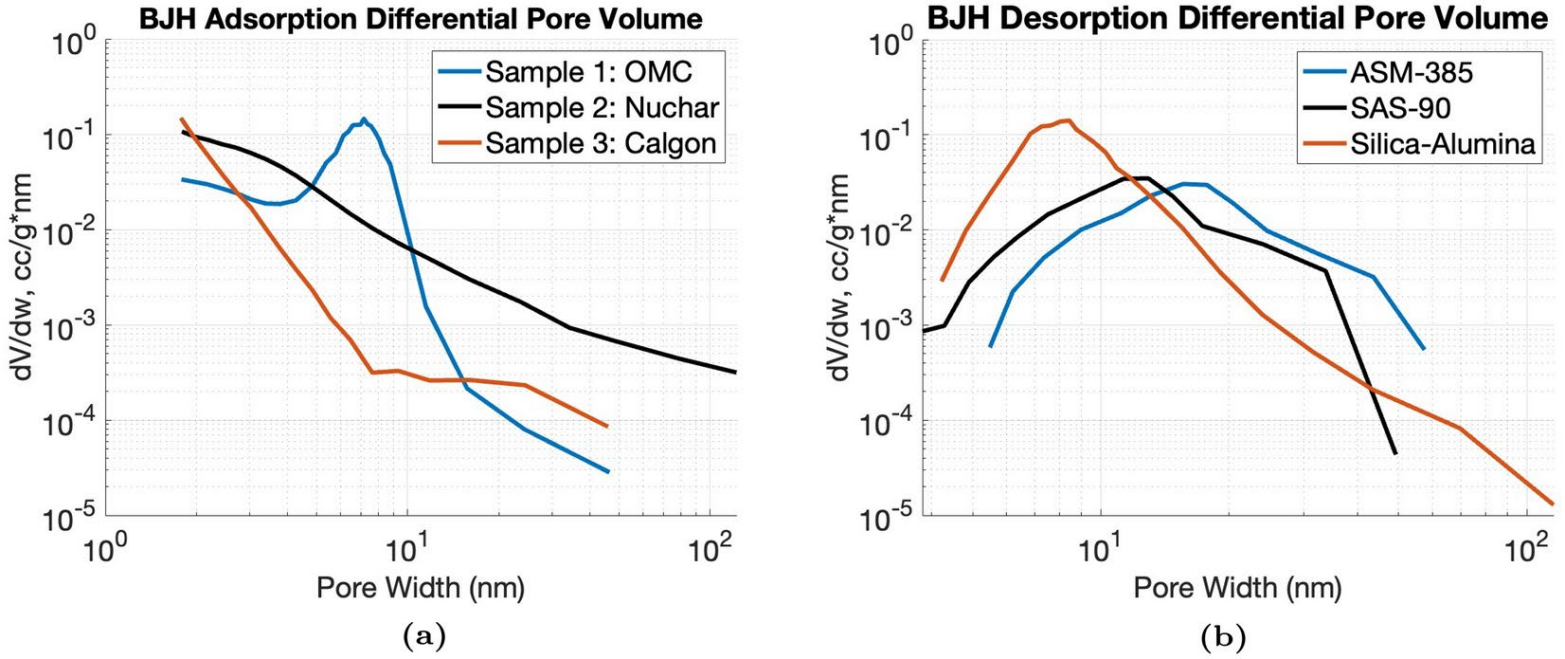
Differential pore volume measurements of the (**a**) carbon and (**b**) alumina samples. The partial pore volume is calculated by integrating from $10 - 120 \, nm$.

As described in Experimental Methods, differential pore volume measurements were taken for each carbon and alumina sample, shown in Figure 7. These curves were over a pore width range of $10 - 120 \, nm$ (or to the maximum pore width) to calculate the partial pore volume (PPV), with results shown in Table 3. For the carbon samples, it is seen that Nuchar has the highest partial pore volume within the range of $10 - 120 \, nm$ and OMC-6-600 and Calgon-PCB are both lower by about an order of magnitude. For the alumina samples, ASM-385 has the highest partial pore volume, followed by SAS-90, and the silica-alumina porosimetry reference has the lowest partial pore volume.

**Table 3:** Total pore volume and partial pore volume within the range of $10-120 \, nm$ for each carbon and alumina sample.

Sample	Total Pore Volume (cc/g)	Partial Pore Volume (cc/g)
OMC-6-600	0.464	0.0168
Nuchar	0.352	0.104
Calgon-PCB	0.0899	0.00711
ASM-385	0.474	0.445
SAS-90	0.408	0.334
Silica-Alumina	0.6699	0.220

#### Carbon and alumina samples: analysis results

**Fig. 8:**
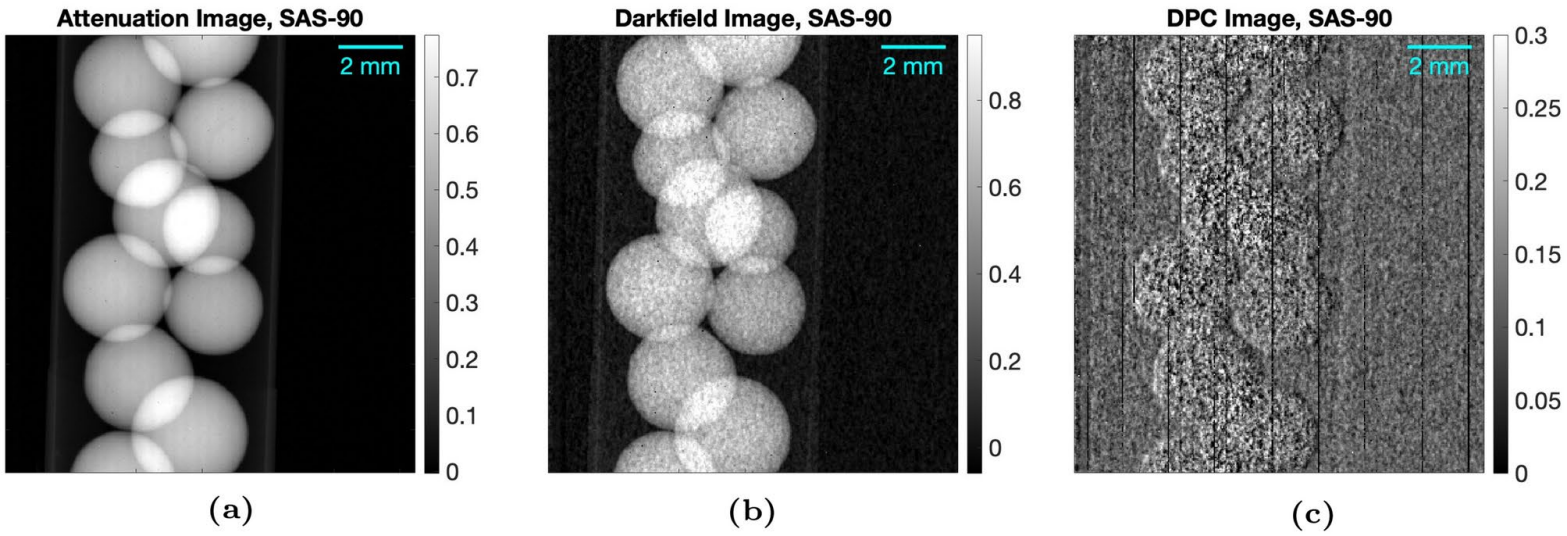
Attenuation (**a**), dark-field (**b**), and differential-phase contrast (**c**) images of SAS-90, taken at a source-to-detector distance of $L = 110 \, cm$, the source-to-grating distance of $L_1 = 20 \, cm$, and grating-to-object distance of $D_{GO} = 40 \, cm$.

Attenuation, dark-field, and differential-phase contrast (DPC) images of each carbon and alumina sample were acquired using the methods described in Experimental Methods. Figure 8 shows example images of the SAS-90 alumina sample. The DPC images are visible for the ASM-385 and SAS-90 alumina samples, where the spheres’ characteristic bright and dark sides are seen. The other samples did not have a significant DPC signal.

**Fig. 9:**
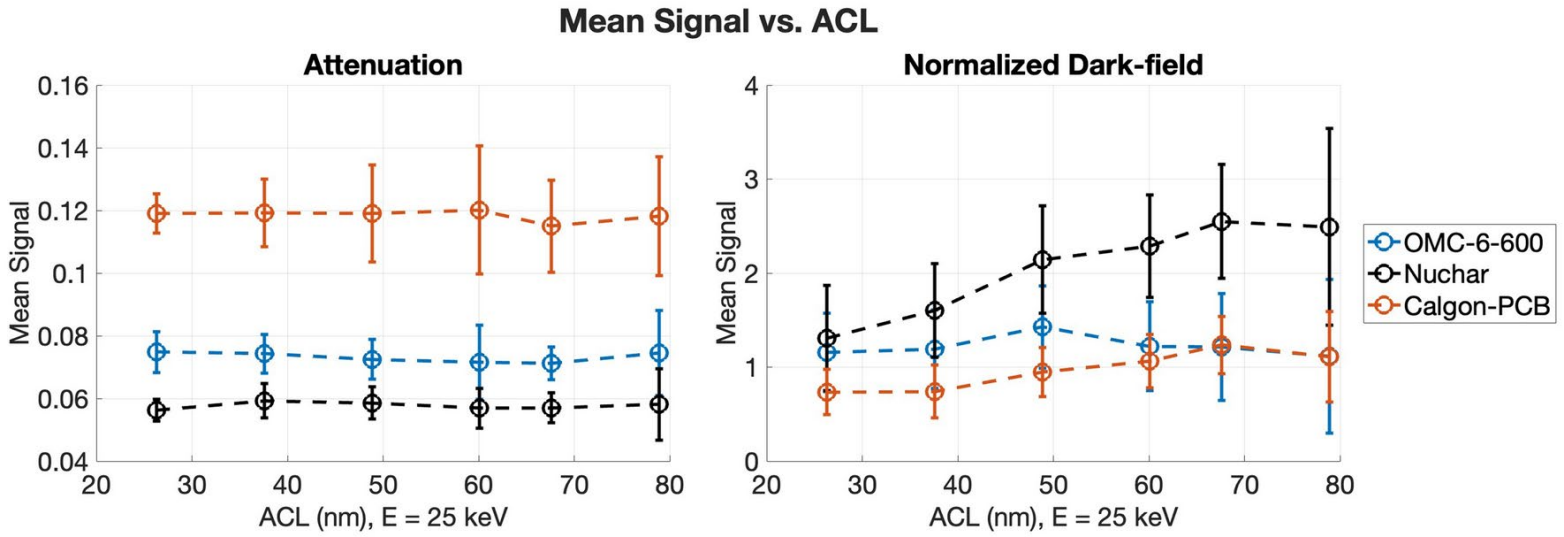
Mean attenuation and mean normalized dark-field signal as a function of ACL for the carbon samples. The ACL was calculated using $E = 25 \, keV$.

**Fig. 10:**
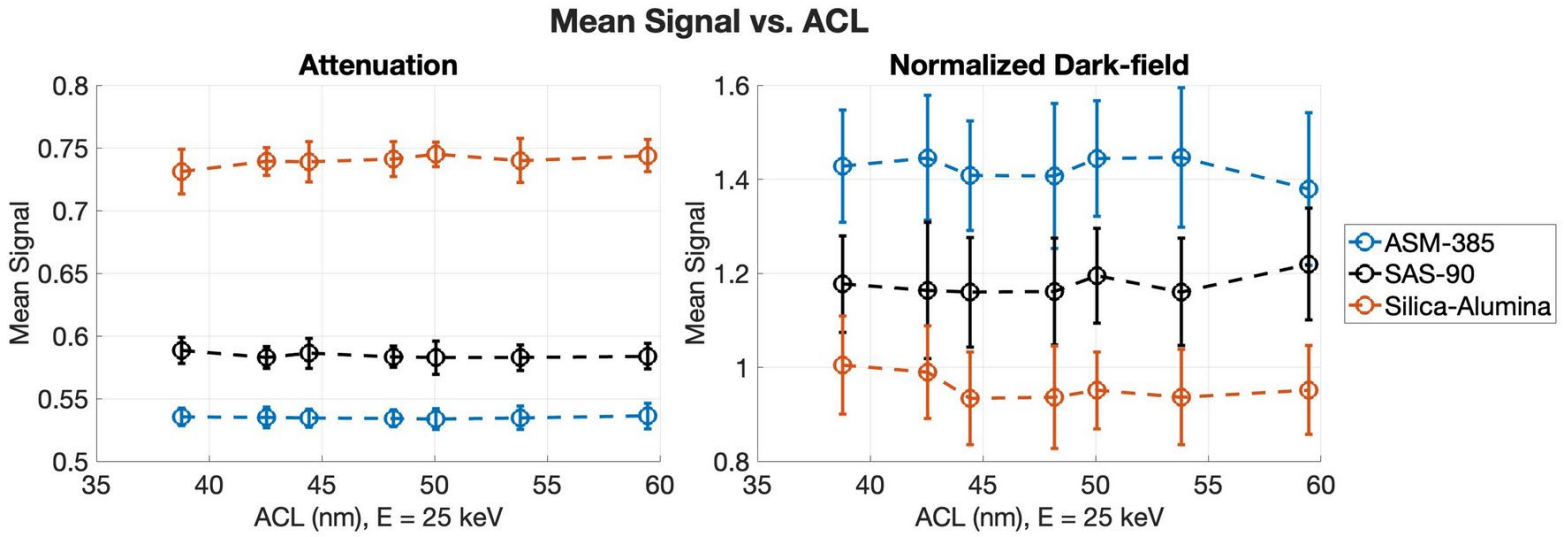
Mean attenuation and mean normalized dark-field signal as a function of ACL for the alumina samples. The ACL was calculated using $E = 25 \, keV$.

For each sample, the mean normalized dark-field signal—the dark-field image divided by the attenuation image—was measured, to be qualitatively compared with the partial pore volume (PPV) of each sample. The mean normalized dark-field signal versus ACL curves are shown in Figures 9 and 10, with similar trends as the PPV measurements shown in Figure 7. For the carbon samples, the normalized dark-field signal is highest for Nuchar, which had the highest partial pore volume. The OMC-6-600 and Calgon-PCB samples both have lower mean signal, with OMC-6-600 being higher on the lower end of the ACL range. This is likely due to the partial pore volume being slightly higher in the OMC-6-600, especially if the pore width range of interest was expanded to include the peak differential pore volume. The mean signal of the Calgon-PCB sample is about the same as the OMC-6-600 sample at the higher end of the ACL range, which may be due to the differential pore volume for Calgon-PCB being larger at the higher end of the pore width range, as seen in Figure 7a. For the alumina samples, the normalized dark-field signal follows the trend of the partial pore volume, with the ASM-385 sample having the highest signal, followed by SAS-90, then the silica-alumina sample.

#### Anchovy images

**Fig. 11:**
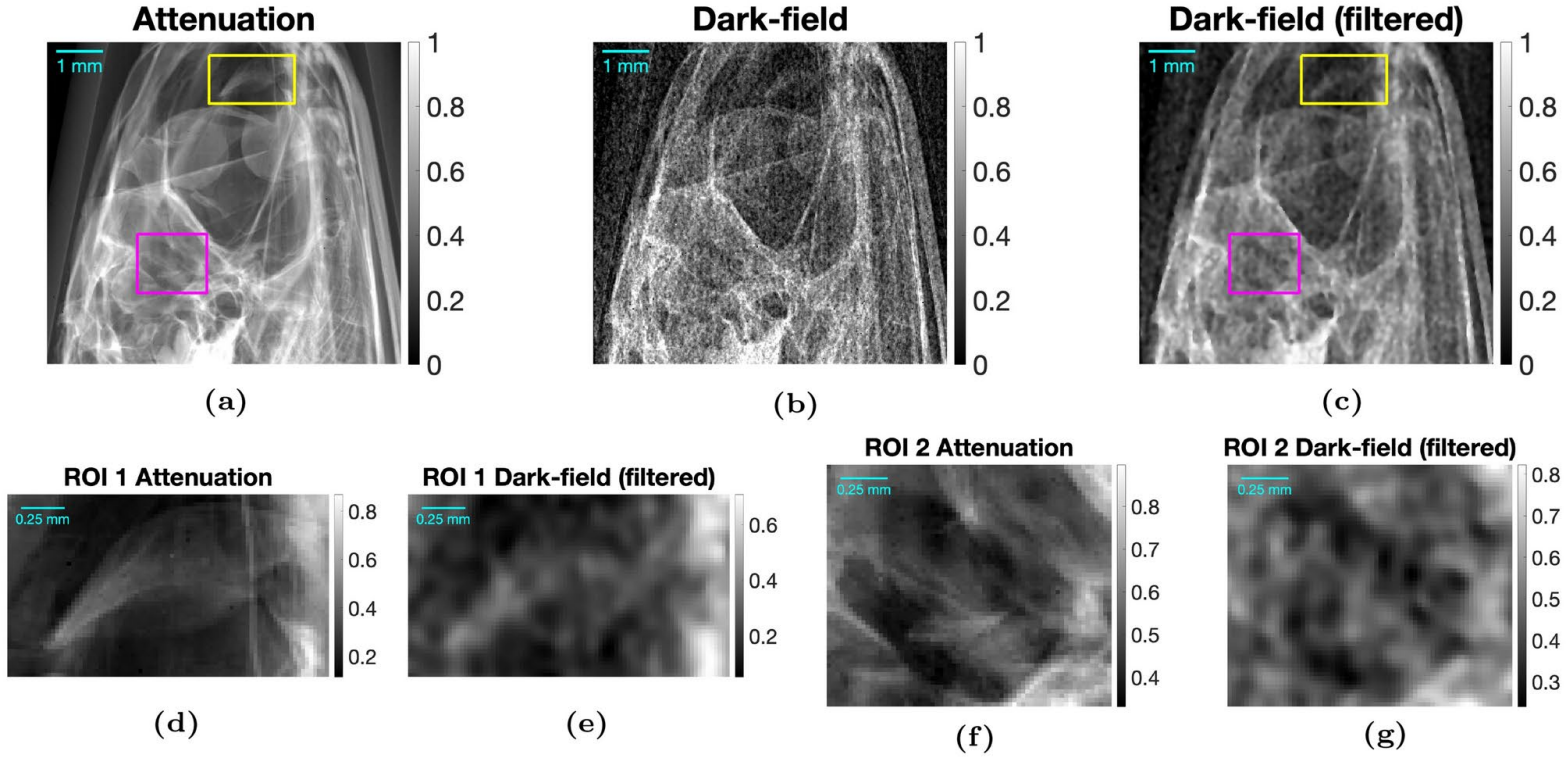
Anchovy images acquired using MPG7. Images were acquired at a source-to-detector distance of $L = 110 \, cm$, a source-to-grating distance of $L_1 = 20 \, cm$, and a grating-to-object distance of $D_{GO} = 13.5 \, cm$, for an autocorrelation length of $ACL = 57.5 \, nm$. The zoomed regions are ROI1 (yellow) and ROI2 (magenta). Note colorbar differences in original and zoomed images.

Images of an anchovy were acquired with MPG7, as explained in Experimental Methods (Anchovy Imaging), shown in Figure 11. At first glance, the dark-field images look like noisier versions of the attenuation images, but on closer inspection, some key differences in structure are seen, which are highlighted. In addition to the attenuation and dark-field images, a filtered dark-field image is shown, where a 3x3 median filter was taken in addition to an anisotropic diffusion filter, with a gradient threshold of 0.2 and 4 iterations. In the filtered image, the noise is reduced and the differences in structure are more visible.

## Discussion

We presented the theory of 2D modulated phase grating fabricated using a Bridge technique. We showed that under realistic detector PSF and source blur, an approximation can be made to reduce computational load by several orders of magnitude. This is known as the $l = 0$ approximation, where all y-harmonics except $l = 0$ are removed. We verified this approximation, and we simulated our experimental setup for measuring the visibility of two RectMPGs, labeled MPG7 and MPG8. Our simulations produced comparable visibility predictions at all distances. This indicates the potential for this model to be used in aiding the design of future MPGI systems.

The theory predicts a drop in visibility at a grating-detector distance of 90-100 cm, but the experimental results show a smaller reduction, especially for MPG8. At these distances, due to fringe magnification, any potential measurement error in the detector point spread function will not have a significant effect. Although the source will show pinhole-type magnification, our investigation shows its impact is not significant, because of its initially small size. The discrepancy could be attributed to uneven phase heights in the gratings, with the deviation being more pronounced in MPG8 compared to MPG7. Additional potential sources of error include improper modeling of the source spectrum or detector response function and diffraction artifacts resulting from the MPGs not exactly following the envelope function, non-rectangular grating bars, or other imperfections in the quality of the gratings. The tolerance for height control has to be determined for future applications. For applications such as iterative reconstruction, calibration procedures may be necessary.

We also imaged several carbon and alumina samples and an anchovy. The differential-phase contrast images of the ASM-385 and SAS-90 samples show the characteristic sides of the alumina spheres. The mean normalized dark-field signal of the carbon and alumina samples was shown to follow the trend of the partial pore volume obtained from BJH adsorption and desorption differential pore volume measurements. The dark-field anchovy images were noisy, but we did observe zones where different structures were visible in the dark-field images compared to the attenuation images. For the visibility experiments, fewer phase steps were used than during the image acquisition of samples, since we were only interested in the average fringe visibility, which should not be biased by the number of phase steps if the signal-to-noise ratio is sufficiently high^38^.

While the gratings used in this study were RectMPGs with phase heights of $(\pi /2, \pi /8)$, we recommend future studies to use $(\pi , 0)$ RectMPGs, as that would produce higher visibility. Additionally, we are actively investigating improvements to the image recovery algorithm, which may lead to improved quality attenuation, dark-field, and DPC images with less phase wraparound and grating shadow artifacts. These artifacts are likely due to multi-harmonic effects and minor motion of grating between taking the reference and sample phase stepping curves. In the future, we believe Controlled Pore Glasses that have near constant pore sizes within the ACL range of our system may be used for verifying results and calibration.

## Conclusion

The modulated phase grating interferometer (MPGI) is a phase sensitive imaging system that simultaneously acquires attenuation, differential-phase contrast, and dark-field images, which show X-ray attenuation, refraction, and small angle scattering, respectively. We have successfully modeled the 2-dimensional MPG fabricated using a Bridge technique, where staggering of the grating bars is present, and we have employed an approximation known as the $l = 0$ approximation to reduce the 2-dimensional calculation to only a single dimension, greatly reducing simulation time. We have shown fringe visibility predictions that are comparable with experimental results and have shown that the model presented has the potential to be used to rapidly iterate when designing future MPGI systems. We have imaged several porous carbon and alumina samples using an MPGI and have shown that the normalized dark-field signal trends well with the partial pore volume of the samples when an appropriate pore width range is used. Lastly, we imaged a dried anchovy and showed multiple regions where unique scattering information is present.

## Supplementary Information

Supplementary Information.

## Data Availability

The datasets used and/or analyzed during the current study are available from the corresponding author on reasonable request.
