# Supplementary material for: Theoretical and experimental analysis of the modulated phase grating X-ray interferometer
Source: Sci Rep. 2024 Nov 5;14:26780. doi: 10.1038/s41598-024-78133-8 (PMC11538336; doi:10.1038/s41598-024-78133-8)
Supplement: Supplementary file 1 — Supplementary Information. [file 41598_2024_78133_MOESM1_ESM.pdf]

Theoretical and Experimental Analysis of the Modulated Phase Grating X-ray Interferometer (Supplementary Material)

Hunter Meyer[1], Joyoni Dey[1,*], Sydney Carr[1], Kyungmin Ham[2], Leslie G. Butler[3], Kerry M. Dooley[4], Ivan Hidrovo[1,5], Markus Bleuel[6], Tamas Varga[7], Joachim Schulz[8,9], Thomas Beckenbach[8], Konradin Kaiser[8]

[1] Department of Physics and Astronomy, Louisiana State University, Baton Rouge, LA, 70803
[2] Center for Advanced Microstructures and Devices, Louisiana State University, Baton Rouge, LA, 70806
[3] Department of Chemistry, Louisiana State University, Baton Rouge, LA, 70803
[4] Cain Department of Chemical Engineering, Louisiana State University, Baton Rouge, LA, 70803
[5] Department of Radiation Therapy, Solón Espinosa Ayala Oncological Hospital, Quito, Ecuador.
[6] Adelphi Technology, Inc., Redwood City, CA, 94063
[7] The Environmental Molecular Sciences Laboratory, Pacific Northwest National Laboratory, Richland, WA
[8] Microworks GmbH, Schnetzlerstr. 9, 76137 Karlsruhe, Germany
[9] Institute of Microstructure Technology, Karlsruhe Institute of Technology, Hermann-von-Helmholtz-Platz 1, D-76344 Eggenstein-Leopoldshafen, Germany.

[*] Corresponding Author: deyj@lsu.edu

S1. The Field Amplitude in 2 Dimensions

For a single system geometry and energy, the angular spectrum method, [1], is used to derive the field amplitude, $U(x)$, for a plane wave source. The Fresnel scaling theorem, [2], is used to scale the field from that of a plane wave source to that of a point-source. Following the angular spectrum method, the angular spectrum immediately following the MPG can be found by taking the Fourier transform of the transmission function, shown in Equation S1. The angular spectrum is shown in Equation S2.

$$\begin{aligned} T(x,y) = 1 + \Bigg\{ (g(x)-1) \Bigg[\sum_n \sum_l \delta(x - np_x)\delta(y - lp_y) \\ + \sum_n \sum_l \delta\left(x - np_x - \frac{p_x}{2}\right) \delta\left(y - lp_y - \frac{p_y}{2}\right) \Bigg] \Bigg\} \star rect\left(\frac{x}{\alpha_x p_x}\right) rect\left(\frac{y}{\alpha_y p_y}\right) \end{aligned} \tag{S1}$$

$$\begin{aligned} A(f_x, f_y, z=0) &= \mathcal{F}\left(T(x,y)\right) \\ A(f_x, f_y, z=0) &= \delta(f_x)\delta(f_y) + \alpha_x\alpha_y \,\mathrm{sinc}(\alpha_x p_x f_x)\,\mathrm{sinc}(\alpha_y p_y f_y)\left(\beta(f_x,f_y) - \gamma(f_x,f_y)\right) \end{aligned} \tag{S2}$$

where,

$$\begin{aligned} \beta(f_x, f_y) = \sum_m \sum_n \sum_l g_m \delta\left(f_x - \frac{m}{W} - \frac{n}{p_x}\right) \delta\left(f_y - \frac{l}{p_y}\right) \\ \times \left(1 + \exp(-j\pi\left(f_x - \frac{m}{W}\right) p_x) \exp(-j\pi f_y p_y)\right) \end{aligned} \tag{S3}$$

$$\gamma(f_x,f_y)=\sum_n\sum_l \delta\left(f_x-\frac{n}{p_x}\right)\delta\left(f_y-\frac{l}{p_y}\right)\Big(1+\exp(-j\pi f_x p_x)\exp(-j\pi f_y p_y)\Big) \tag{S4}$$

The angular spectrum can then be propagated to the detector,

$$A(f_x,f_y,z)=A(f_x,f_y,z=0)\,e^{jkz}\,\exp\left(-j\pi\lambda z(f_x^2+f_y^2)\right) \tag{S5}$$

And the field is calculated,

$$\begin{aligned} U(x,y,z)&=\mathcal{F}^{-1}\left(A(f_x,f_y,z)\right)\\ &=e^{jkz}(1+U_1(x,y,z)-U_2(x,y,z)) \end{aligned} \tag{S6}$$

$U_1(x,y,z)$ and $U_2(x,y,z)$ are derived from the propagation of $\beta(f_x,f_y)$ and $\gamma(f_x,f_y)$, respectively. The x- and y-components of each term can be represented separately using matrix multiplication,

$$\begin{bmatrix}U_1(x,y,z)\\U_2(x,y,z)\end{bmatrix}=\begin{bmatrix}U_1^{Ax}(x,z) & U_1^{Bx}(x,z)\\U_2^{Ax}(x,z) & U_2^{Bx}(x,z)\end{bmatrix}\times\begin{bmatrix}U^{Ay}(y,z)\\U^{By}(y,z)\end{bmatrix} \tag{S7}$$

where the x-components are

$$U_1^{Ax}(x,z)=\sum_m\sum_n A_{1x}(m,n,z)\exp\left(j2\pi x\left(\frac{m}{W}+\frac{n}{p_x}\right)\right) \tag{S8}$$

$$U_1^{Bx}(x,z)=\sum_m\sum_n B_{1x}(m,n,z)\exp\left(j2\pi x\left(\frac{m}{W}+\frac{n}{p_x}\right)\right) \tag{S9}$$

$$U_2^{Ax}(x,z)=\sum_m\sum_n A_{2x}(n,z)\exp\left(j2\pi x\frac{n}{p_x}\right) \tag{S10}$$

$$U_2^{Bx}(x,z)=\sum_m\sum_n B_{2x}(n,z)\exp\left(j2\pi x\frac{n}{p_x}\right) \tag{S11}$$

$$A_{1x}(m,n,z)=\alpha_x g_m\exp\left(-j\pi\lambda z\left(\frac{m}{W}+\frac{n}{p_x}\right)^2\right)\mathrm{sinc}\left(\alpha_x p_x\left(\frac{m}{W}+\frac{n}{p_x}\right)\right) \tag{S12}$$

$$A_{2x}(n,z)=\alpha_x\exp\left(-j\pi\lambda z\left(\frac{n}{p_x}\right)^2\right)\mathrm{sinc}\left(\alpha_x n\right) \tag{S13}$$

$$B_{1x}(m,n,z)=A_{1x}(m,n,z)\exp\left(-j\pi n\right) \tag{S14}$$

$$B_{2x}(n,z)=A_{2x}\exp\left(-j\pi n\right) \tag{S15}$$

and the y-components are

$$U_{Ay}(y,z)=\sum_l A_y(l,y,z)\exp\left(j2\pi y\frac{l}{p_y}\right) \tag{S16}$$

$$U_{By}(y,z)=\sum_l B_y(l,y,z)\exp\left(j2\pi y\frac{l}{p_y}\right) \tag{S17}$$

$$A_y(l,z)=\alpha_y\exp\left(-j\pi\lambda z\left(\frac{l}{p_y}\right)^2\right)\mathrm{sinc}\left(\alpha_y l\right) \tag{S18}$$

$$B_y(l,z)=A_y(l,y,z)\exp\left(-j\pi l\right) \tag{S19}$$

Finally, the Fresnel scaling theorem can be applied by scaling x, y, and z by the point-source magnification factor, $M=\frac{L_1+z}{L_1}$,

$$x \longrightarrow \frac{x}{M}$$
$$y \longrightarrow \frac{y}{M}$$
$$z \longrightarrow \frac{z}{M}$$

It should be noted that there are additional phase and amplitude multiplicative factors introduced by the Fresnel scaling theorem that we do not consider for the purposes of this study, since the phase factors disappear when calculating the intensity and the amplitude factors do not affect the visibility.

S2. The Field Intensity and the l = 0 Approximation

The field intensity can be calculated as simply the square of the amplitude.

$$I(x, y, z) = |U(x, y, z,)|^2 = U(x, y, z)U^*(x, y, z) \tag{S20}$$

Since the detector will blur the y-harmonics due to the relatively small p_y, the detector intensity will be well approximated by the $l = 0$ harmonic, analogous to the $n = 0$ approximation present in [3]. To simplify this, we will represent the field amplitude from Equation S6 using only the y-harmonics (ignoring the e^{ikz} which disappears in the intensity). The $l = 0$ approximation must be taken in the *intensity*, not the amplitude, but representing the field amplitude in this way will simplify the calculations. The $l = 0$ harmonic has the primary benefit of greatly reducing the required computations, since the intensity is reduced to 1 dimension while maintaining the effects caused by the staggering of the grating bars.

$$U(x, y, z) = 1 + \sum_l c(l, x, z) \exp\left(j2\pi y \frac{l}{Mp_y}\right) \tag{S21}$$

where,

$$c(l, x, z) = U_{Ax}(x, z)A_y(l) + U_{Bx}(x, z)B_y(l) \tag{S22}$$
$$U_{Ax}(x, z) = U_1^{Ax}(x, z) - U_2^{Ax}(x, z) \tag{S23}$$
$$U_{Bx}(x, z) = U_1^{Bx}(x, z) - U_2^{Bx}(x, z) \tag{S24}$$

The intensity can then be calculated:

$$I(x, y, z) = 1 + \sum_l \Big[c(l, x, z) + c^*(-l, x, z) + d(l, x, z)\Big] \exp\left(j2\pi y \frac{l}{Mp_y}\right) \tag{S25}$$

where,

$$d(l, x, z) = c(l, x, z) \star c^*(-l, x, z) \tag{S26}$$

The $l = 0$ approximation is then taken,

$$I(x, y, z) \approx 1 + c(0, x, z) + c^*(0, x, z) + d(0, x, z) \tag{S27}$$

$c(0, x, z)$ and $c^*(0, x, z)$ are easy to compute by recognizing that $A_y(0) = B_y(0) = \alpha_y$, whereas $d(0, x, z)$ can be easily computed,

$$\begin{aligned} d(0, x, z) = {} & U_{Ax}U_{Ax}^* \sum_{l'} s(l') + U_{Ax}U_{Bx}^* \sum_{l'} s(l') \exp(-j\pi l') \\ & + U_{Ax}^*U_{Bx} \sum_{l'} s(l') \exp(j\pi l') + U_{Bx}U_{Bx}^* \sum_{l'} s(l') \end{aligned} \tag{S28}$$

where,

$$s(l) = A_y(-l) \star A_y^*(-l) = \alpha_y^2 \,\mathrm{sinc}\,(-\alpha_y l)^2 \tag{S29}$$

The $l = 0$ approximation greatly reduces the computation required and is only valid if $M * p_y$ is significantly less than the pixel size. Notably, this does not fully remove the effect of staggering on the fringes. The intensity profile still depends on α_y, meaning the fringe visibility will also depend on it.
